# Longitudinal Validation of Clinical Care Pathways for Metabolic Dysfunction–Associated Steatotic Liver Disease in a Prospective Cohort of Individuals With Type 2 Diabetes

**DOI:** 10.1016/j.gastha.2026.101022

**Published:** 2026-05-27

**Authors:** Jonathan Dounel, Luis Antonio Díaz, Ricki Bettencourt, Federica Tavaglione, Egbert Madamba, Lisa Richards, Rohit Loomba, Veeral Ajmera

**Affiliations:** 1 MASLD Research Center, Division of Gastroenterology and Hepatology, University of California at San Diego, San Diego, California; 2 Departamento de Gastroenterología, Escuela de Medicina, Pontificia Universidad Católica de Chile, Santiago, Chile

**Keywords:** MASH, Diabetes, Screening, Risk Stratification

## Abstract

**Background and Aims:**

The American Gastroenterological Association (AGA) Clinical Care Pathway provides a tiered, noninvasive algorithm for fibrosis risk stratification in populations at high risk for metabolic dysfunction–associated steatotic liver disease (MASLD). We assessed the 2-year longitudinal performance of the AGA pathway using magnetic resonance elastography (MRE) as the reference.

**Methods:**

This prospective cohort study enrolled adults aged 50–79 years with type 2 diabetes mellitus from ambulatory care clinics between the dates of February 2016 and February 2025. Participants underwent a standardized clinical research visit with Fibrosis-4 index (FIB-4), vibration-controlled transient elastography (VCTE), and MRE at baseline and at a 2-year interval at the UCSD MASLD Research Center.

**Results:**

Of 626 participants with a baseline assessment, 209 had longitudinal follow-up assessment and were included in the study. The prevalence of MASLD was 69.9%, and 19.0% had significant fibrosis at baseline (MRE ≥3.30 kPa). Applying the AGA pathway of FIB-4 and VCTE, the false negative rate (low risk by pathway with MRE ≥3.30 kPa) at baseline was 7% with 17.6% of participants qualifying for specialty referral. At 2-year follow-up, the false negative rate decreased to 3% and an additional 7%, respectively qualified for specialty referral. Applying a FIB-4 cut point of 1.0 decreased the false negative rate to 0%; however, the number of patients requiring VCTE increased by 54% over 2-years.

**Conclusion:**

Longitudinal reassessment of patients initially classified as low risk by the AGA Clinical Care Pathway substantially reduced misclassification of significant fibrosis, while maintaining a low rate of specialty referral. These findings support serial re-evaluation as a key component of noninvasive fibrosis risk stratification in at-risk populations.

## Introduction

Metabolic dysfunction–associated liver disease (MASLD), formerly known as nonalcoholic fatty liver disease, has become a dominant contributor to chronic liver disease worldwide.^1^, ^2^, ^3^ Individuals with type 2 diabetes mellitus (T2DM) are particularly vulnerable, with a high prevalence of MASLD and an increased risk of progression to advanced fibrosis, cirrhosis, and hepatocellular carcinoma.^4^, ^5^, ^6^, ^7^, ^8^, ^9^, ^10^ These outcomes often occur silently, making early and accurate identification of fibrosis critical to reducing liver-related morbidity and mortality in this population.

Academic societies, including the American Gastroenterological Association (AGA), the American Association for the Study of Liver Diseases, and other international societies, have developed structured, noninvasive pathways to guide MASLD risk stratification for at-risk populations.^11^, ^12^, ^13^, ^14^, ^15^ These recommendations are focused on identifying patients with fibrosis who are most likely to benefit from hepatology referral or therapeutic intervention and minimize unnecessary evaluations for low-risk individuals. Thus, they recommend a tiered approach beginning with the Fibrosis-4 index (FIB-4), followed by either vibration-controlled transient elastography (VCTE) or the Enhanced Liver Fibrosis test for those with indeterminate scores. For those identified as low risk, reassessment with these strategies is recommended within 2 to 3 years.

To evaluate the diagnostic performance of this clinical pathway, an external, high-fidelity reference standard is essential to determine the true prevalence of fibrosis across risk strata. Magnetic resonance elastography (MRE), which is not incorporated into current care pathways, offers a highly accurate, noninvasive assessment of liver stiffness, with excellent specificity and positive predictive value (PPV) for advanced fibrosis.^16^, ^17^, ^18^ A prior cross-sectional validation study showed that applying the pathway in a T2DM cohort led to effective triage, limited the number of referrals to specialty care, and resulted in a low false negative rate when benchmarked against MRE.^19^ However, clinical guidance recommends repeating noninvasive assessment every 2 to 3 years in patients with lowest risk, yet few data exist to support the longitudinal performance of these pathway-based triage strategies.^11^^,^^12^ To address this gap, we conducted a prospective longitudinal study of adults with T2DM who underwent baseline and 2-year follow-up research assessment with MRE. Our primary aim was to evaluate how repeat use of the clinical care pathway performs over time in detecting significant fibrosis.

## Materials and Methods

### Study Design

In this longitudinal study, we prospectively enrolled adults with T2DM from primary care and endocrinology clinics in the greater San Diego area, as described previously.^4^ All participants were invited to undergo a standardized research visit with clinical history and fasting laboratory parameters, VCTE, magnetic resonance imaging (MRI)–based proton density fat fraction (PDFF) and MRE followed by repeat assessment after 2 years between the dates of February 2016 and February 2025 at the UCSD MASLD Research Center. Patients provided written informed consent prior to enrolling in the study, and the study was approved by the UCSD Institutional Review Board (IRB Protocol #160231). This study was conducted under Good Clinical Practice guidelines and the Declaration of Helsinki. The authors had access to the data and participated in analyses and drafting the manuscript.

### Study Population

Adults aged 50–79 years with a diagnosis of T2DM, defined according to American Diabetes Association criteria, were eligible for enrollment. This age range was selected as part of the parent prospective diabetes cohort to enrich for individuals at higher risk of advanced fibrosis,^4^ consistent with prior studies demonstrating increased prevalence of fibrotic MASLD among older adults with T2DM.^20^ T2DM diagnosis required either an elevated fasting plasma glucose, abnormal oral glucose tolerance test, hemoglobin A1c ≥ 6.5%, or symptoms of hyperglycemia with a random plasma glucose ≥200 mg/dL. All participants were required to provide written informed consent and demonstrate willingness and ability to comply with study procedures. All included patients had at least FIB-4 and MRE assessments. Participants were excluded for known or suspected alternative causes of liver disease, including significant alcohol use, viral hepatitis (B or C), autoimmune hepatitis or cholestatic liver disease, Wilson disease, alpha-1 antitrypsin deficiency, hemochromatosis, drug-induced liver injury, or biliary obstruction. Additional exclusion criteria included evidence or history of cirrhosis or portal hypertension, prior bariatric surgery (including Roux-en-Y gastric bypass or vertical sleeve gastrectomy), recent use of steatogenic medications, pregnancy or nursing, life expectancy <5 years, and contraindications to MRI.

### Clinical Assessment and Laboratory Tests

All included patients underwent 2 standardized clinical research evaluations, one at baseline and one at 2-year interval. These evaluations included a detailed history and a physical examination, which included vital signs, height, weight, and anthropometric measurements, performed by a trained investigator. Body mass index (BMI) was defined as the body weight (in kilograms) divided by height (in meters) squared. Alcohol consumption was assessed through standardized validated tools, Alcohol Use Disorders Identifications Test and the Skinner^21^ questionnaire, a 20-item survey derived from the Diagnostic and Statistical Manual of Mental Disorders criteria and designed to identify hazardous and dependent alcohol use. Patients underwent the following biochemical tests after a minimum of an 8 hour fast: glucose, albumin, hemoglobin A1c, alanine aminotransferase (ALT), aspartate aminotransferase (AST), total bilirubin, alkaline phosphatase, fasting lipid panel, platelets, insulin, international normalized ratio. Patients also underwent VCTE and MRE. At the 2-year follow-up, all included participants had an updated repeat clinical research examination, including standardized history, fasting biochemical tests, and repeat FIB-4, VCTE, and MRE.

### Clinical Care Pathway Validation

FIB-4 was calculated as described previously and patients with a FIB-4 < 1.3 were considered low risk.^22^ Patients with FIB-4 1.3 to 2.67 were indeterminate risk and >2.67 as high risk. The diagnostic performance of further risk assessment by liver stiffness on VCTE by FibroScan (Echosens) was assessed for patients with indeterminate FIB-4.^23^ All exams were performed by an experienced technician after a minimum fast of 4 h as recommended. During patient breath holding, a minimum of 10 repeated valid measurements, assessed automatically by the FibroScan system, was performed. All participants were first scanned using the M probe (3.5 MHz). If indicated upon initial assessment, participants were rescanned using the XL probe (2.5 MHz). Examinations that did not meet quality criteria, defined as an interquartile range (IQR)/median ratio of >0.30 when the median liver stiffness was ≥7.1 kPa were excluded. VCTE <8 kPa was considered low risk, 8 to 12 kPa indeterminate risk and ≥12 kPa high risk. At 2-year interval, a repeat assessment was completed and the same cut points for FIB-4 and VCTE were evaluated.

### Magnetic Resonance Imaging

Participants included in this study underwent a noncontrast MRI exam with liver fat quantification and liver stiffness assessment using PDFF and MRE, respectively at baseline and follow-up. Imaging was performed using a 3T research scanner (GE Signa EXCITE HDxt; GE Healthcare, Waukesha, WI) and liver stiffness data were obtained using two-dimensional MRE at 60 Hz. Acquired MR images were interpreted by an experienced radiologist to quantify liver fat and stiffness, as previously described.^16^^,^^24^^,^^25^

### Outcome Measures

The primary outcome was to determine the false negative rate, at baseline and 2-year follow-up, defined as the percentage of patients classified as low risk who had significant fibrosis on MRE assessment, defined as MRE ≥3.3 kPa.^17^^,^^26^ A secondary outcome of this study was to determine the diagnostic performance of noninvasive tests (NITs), including their sensitivity and specify for significant fibrosis.

### Statistical Analysis

For patient characteristics, continuous variables were presented as mean (standard deviation [SD]) or as median (IQR) if the distribution was not normal. The diagnostic performance of FIB-4 and VCTE were expressed as sensitivity, specificity, PPV, and negative predictive value (NPV) at prespecified cut points. All statistical analyses were performed using SAS (version 9.4; SAS Institute), and a two-tailed *P* value less than 0.05 was considered statistically significant. As part of the sensitivity analysis, we evaluated applying a lower FIB-4 cut point of 1.0 to define low risk, where a FIB-4 of 1.0 to 2.67 would fall in the indeterminate risk range. We also applied a lower MRE threshold of 3.14 kPa to define significant fibrosis to evaluate the false negative rate at baseline and follow-up.

## Results

### Characteristics of the Study Population

**Figure 1:**
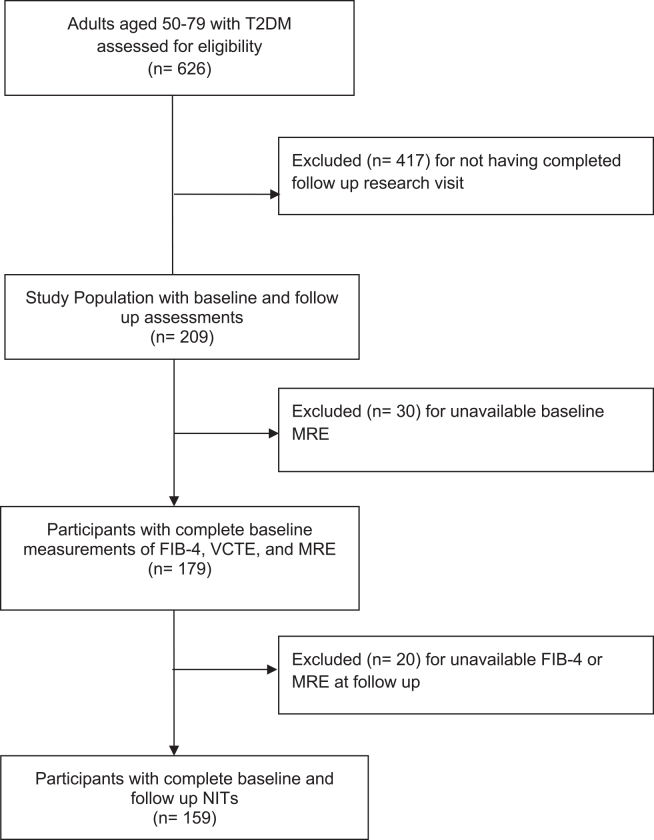
Consort diagram.

Of 626 participants with T2DM assessed for eligibility at baseline, 209 individuals had 2-year follow-up and were included in the study, and 179 (85.6%) of them had valid FIB-4, VCTE, and MRE measurements at baseline (Figure 1); only 7 (1.1%) of participants were excluded due to invalid VCTE measurements. Participants had a mean (±SD) age of 64.3 (±7.9) years and were predominantly female (60.3%) (Table 1). The majority identified as White (49.3%) and 29.8% were Hispanic. The mean BMI (±SD) was 31.2 (±4.9) kg/m2. The cohort had a high prevalence of cardiometabolic comorbidities, with hypertension affecting 130 (62.2%) of participants and hyperlipidemia affecting 123 (58.9%). Baseline glycemic control was moderate, with a median (IQR) hemoglobin A1c (HbA1c) of 6.8% (1.5); HbA1c levels were slightly higher among participants with significant fibrosis compared to those without (7.1% vs 6.6%). The prevalence of MASLD (MRI-PDFF ≥5% after exclusion of other liver diseases) was 69.9%, and 19.0% had significant fibrosis at baseline (MRE ≥3.30 kPa). The mean (±SD) liver fat on MRI-PDFF was 10.5% (±7.7) and mean liver stiffness on MRE was 2.8 kPa (±1.3). FIB-4 values of <1.3, 1.3 to 2.67, and ≥2.67 were present in 101 (48.3%), 93 (44.5%), and 15 (7.2%) of patients, respectively. VCTE values <8 kPa, 8 to 12 kPa and ≥12 kPa were present in 138 (68.3%), 34 (16.8%), and 30 (14.9%) of patients, respectively.

**Table 1:** Clinical, Demographic, and Imaging Characteristics by Advanced Fibrosis Status of Study Population at Baseline

	Total N = 209	Significant fibrosis (MRE ≥3.3 kPa) N = 34	No significant fibrosis (MRE <3.3 kPa) N = 145
Demographic and clinical			
Age in years, mean (SD)	64.3 (7.9)	63.6 (7.2)	64.4 (8.1)
Female, n (%)	126 (60.3%)	26 (76.5%)	87 (60.0%)
BMI (kg/m^2^), mean (SD)	31.2 (4.9%)	31.0 (4.0)	30.4 (4.3)
Obesity (BMI ≥30 kg/m^2^)	125 (59.8%)	24 (70.6%)	78 (53.8%)
Race			
White, n (%)	101 (49.3%)	13 (39.4%)	68 (47.9%)
Hispanic, n (%)	61 (29.8%)	11 (33.3%)	46 (32.4%)
Asian, n (%)	30 (14.6%)	7 (21.2%)	20 (14.1%)
Other, n (%)	13 (6.3%)	2 (6.1%)	8 (5.6%)
Duration of DM (years), median (IQR)	8.0 (12.0)	10 (9)	7 (12)
Hypertension, n (%)	130 (62.2%)	19 (55.9%)	88 (60.7%)
Hyperlipidemia, n (%)	123 (58.9%)	18 (52.9%)	86 (59.3%)
Metabolic syndrome, n (%)	119 (65.8%)	22 (68.8%)	77 (63.1%)
Biochemical profile			
HbA1c (%), median (IQR)	6.8 (1.5)	7.1 (2)	6.6 (1.5)
HOMA-IR	4.8 (5.3)	8.7 (7.3)	4.3 (3.8)
AST (U/l), median (IQR)	26 (17)	43 (30)	25 (13)
ALT (U/l), median (IQR)	29 (25)	40 (45)	26 (20)
Alkaline phosphatase (U/l), median (IQR)	76 (34)	81.5 (36)	71 (33)
Total bilirubin (mg/dl), median (IQR)	0.5 (0.2)	0.5 (0.3)	0.5 (0.2)
Albumin (g/dl), median (IQR)	4.5 (0.3)	4.5 (0.4)	4.5 (0.4)
Triglycerides (mg/dl), median (IQR)	143 (80)	130.5 (99)	143 (73)
HDL (mg/dl), median (IQR)	45.5 (16)	43.5 (19)	46 (14)
LDL (mg/dl), median (IQR)	84 (45)	79 (40)	85 (45)
Platelet count (10^9^/L), median (IQR)	245 (84)	216.5 (90)	258 (89)
INR, median (IQR)	1 (0.1)	1.1 (0.1)	1 (0.1)
Blood based scores and markers			
FIB-4, median (IQR)	1.3 (0.8)	1.9 (1.3)	1.2 (0.6)
NAFLD fibrosis score, median (IQR)	−0.4 (1.5)	−0.1 (1.3)	−0.7 (1.4)
Imaging			
CAP (dB/m), mean (SD)	313 (52.2)	313.8 (47.5)	309.7 (52.6)
VCTE (kPa), mean (SD)	7.9 (6.9)	13.1 (4.4)	5.8 (2.3)
MRI-PDFF (%), mean (SD)	10.5 (7.7)	11.0 (7.5)	10.4 (7.8)
MRE (kPa), mean (SD)	2.8 (1.3)	4.6 (1.8)	2.3 (0.4)

*T*-test performed on continuous variables presented as mean (SD), Wilcoxon rank-sum test performed on all other continuous variables. Significant fibrosis defined as MRE ≥3.30 kPa.

ALT, alanine aminotransferase; AST, aspartate aminotransferase; BMI, body mass index; CAP, controlled attenuation parameter; DM, diabetes mellitus; FIB-4, Fibrosis-4 index; HbA1c, hemoglobin A1c; HDL, high-density lipoprotein; HOMA-IR, homeostatic model assessment for insulin resistance; INR, international normalized ratio; IQR, interquartile range; LDL, low-density lipoprotein; MRI-PDFF, magnetic resonance imaging–based proton density fat fraction; MRE, magnetic resonance elastography; NAFLD, nonalcoholic fatty liver disease; SD, standard deviation.

We compared baseline characteristics between participants who completed follow-up (n = 209) and those with baseline assessment only (n = 417) (Supplementary Table 1). Baseline demographic, clinical, biochemical, and imaging characteristics were largely similar between groups. The notable statistically significant difference observed was race distribution (*P* < .0001). Participants who did not complete follow-up were more likely to be Hispanic (46.9% vs 29.8%) and less likely to be non-Hispanic White (30.8% vs 49.3%) compared with those who completed follow-up. There were no significant differences in age, sex, BMI, metabolic comorbidities, or baseline noninvasive fibrosis markers including FIB-4, VCTE, and MRE.

### Diagnostic Performance of Fibrosis Index Based on the 4 Factor and Vibration-Controlled Transient Elastography in the Study Population

The diagnostic accuracy of FIB-4 for significant fibrosis using a cut point of 1.3 yielded a sensitivity of 71%, specificity of 57%, PPV of 28%, and NPV of 89% (Table 2). Lowering the cut point to 1.0 increased sensitivity to 88% and decreased specificity and PPV to 28% and 22%, respectively. The diagnostic accuracy of VCTE for significant fibrosis using the cut point of 8 kPa yielded a sensitivity of 94%, specificity of 86%, PPV of 61%, and NPV of 98%. Using a higher cut point of 12 kPa yielded a sensitivity of 58%, specificity of 98%, PPV of 86%, and NPV of 91%.

**Table 2:** Diagnostic Accuracy of VCTE, FIB-4 for Significant Fibrosis Assessed by MRE ≥3.3 kPa at Baseline

	AUROC	Cutoff	Sensitivity (%)	Specificity (%)	PPV (%)	NPV (%)
VCTE	0.89 (0.85-0.95)	8 kPa	94	86	61	98
	0.78 (0.69-0.86)	12 kPa	58	98	86	91
FIB-4	0.58 (0.52-0.65)	1.0	88	28	22	91
	0.64 (0.55-0.73)	1.3	71	57	28	89

### Performance of the Clinical Pathway

**Figure 2:**
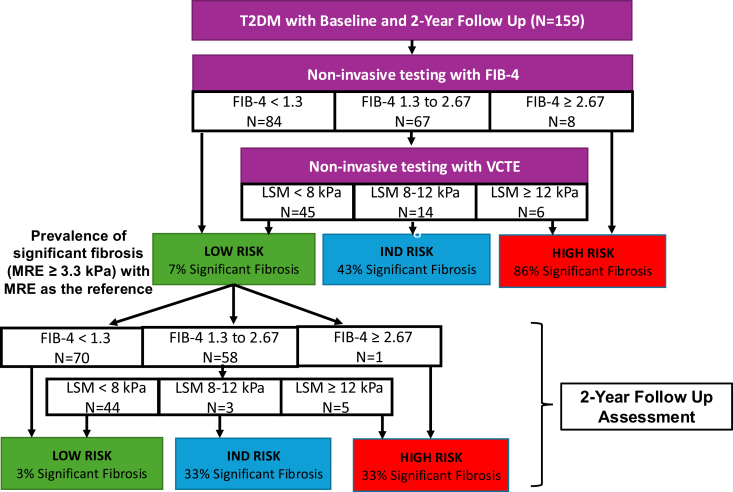
Performance of AGA Clinical Pathway in adults with T2DM with MRE as the reference, at baseline and at 2-year follow-up.

Of the 209 participants at baseline assessment, 159 (76.1%) participants had MRE assessments at baseline and follow-up. Among these 159 participants, 52.8% were classified as low risk by FIB-4, 42.1% as indeterminate, and 5.0% as high risk. Among those with indeterminate FIB-4, 67.2% had VCTE <8 kPa (low risk), 20.9% had VCTE 8 to 12 kPa (indeterminate risk), and 8.9% had VCTE ≥12 kPa (high risk) (Figure 2). The prevalence of significant fibrosis (defined as MRE ≥3.3 kPa) in the low-risk group, defined as the false negative rate, was 7%. The prevalence of significant fibrosis was 43% among indeterminate-risk and 86% among high-risk participants. The sum of indeterminate- and high-risk groups requiring specialty referral is 28 (17.6%) of the total population.

There was a total number of 129 participants in the low-risk group that had reassessment in 2 years as described previously with FIB-4, VCTE, and MRE. At 2-year reassessment, 88.3% of participants classified as low risk at baseline remained low risk, while 7% were reclassified as indeterminate and 4.7% as high risk. The prevalence of significant fibrosis (MRE ≥3.3 kPa) was 3% in the low-risk group, 33% in the indeterminate-risk group, and 33% in the high-risk group. Based on AGA criteria incorporating FIB-4 and VCTE, 9 (7%) additional individuals met thresholds for specialty referral.

### Changes From Baseline to Follow-Up Assessment

We evaluated changes in noninvasive tests among participants classified as low risk for fibrosis at baseline (Table 3). The mean (SD) change in FIB-4 was 0.08 (0.37) and mean (SD) change in VCTE was 0 (3.37) kPa. Mean (SD) change in MRI-PDFF and MRE were −1.93 (5.79) and 0.03 (0.49), respectively. Median change in AST and ALT were 0 (10) and −2 (16) U/L, respectively. The indeterminate and high-risk groups similarly experienced only small changes in NITs over the 2-year period.

**Table 3:** Delta Change, per Persons, Categorized by Risk From Baseline Assessment

	Low risk N = 161	Indeterminate risk N = 19	High risk N = 26
Δ FIB-4, mean (SD)	0.08 (0.37)	0.08 (0.69)	0.21 (1.57)
Δ VCTE (kPa), mean (SD)	0 (3.37)	−0.14 (6.18)	2.01 (16.59)
Δ MRI-PDFF (%), mean (SD)	−1.93 (5.79)	−3.46 (7.88)	−0.37 (4.00)
Δ MRE (kPa), mean (SD)	0.03 (0.49)	0.25 (0.89)	−0.39 (2.88)
Δ AST (U/l), median (IQR)	0 (10)	−7.5 (13)	−6 (22)
Δ ALT (U/l), median (IQR)	−2 (16)	−9 (16)	−10 (15)

### Follow-Up of Patients With Baseline False Negative Fibrosis Assessment

**Figure 3:**
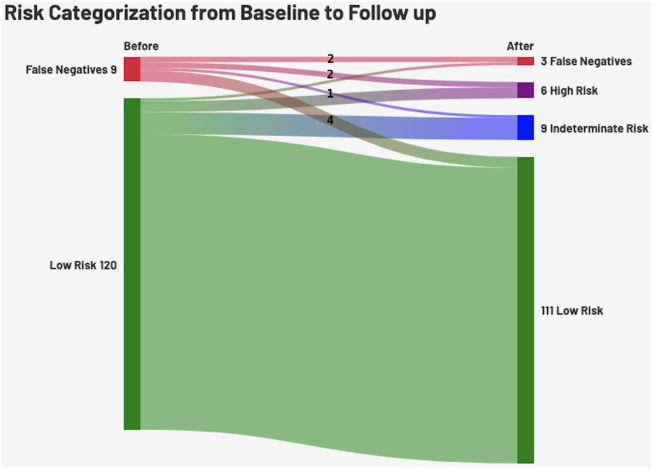
Risk categorization from baseline to follow-up.

At baseline, 9/129 participants (7%) were classified as low risk but had significant fibrosis on MRE (≥3.3 kPa), representing false negatives. Of these, 8/9 (88.9%) had FIB-4 scores <1.3 and 1/9 (11.1%) had FIB-4 ≥ 1.3 and VCTE <8 kPa. At 2-year follow-up, 2 of these participants had FIB-4 increase to >1.3 and were appropriately triaged into high risk with evidence of significant fibrosis. One participant had increased FIB-4 and VCTE now consistent with indeterminate risk. Three participants maintained low FIB-4 but now no longer had evidence of significant fibrosis on MRE. One participant had increased FIB-4 but low VCTE and was appropriately triaged into low risk. Two participants remained false negatives, with FIB-4 <1.3 and persistent MRE evidence of significant fibrosis (Figure 3). One participant had MRE with evidence of significant fibrosis but was newly falsely triaged into low risk by FIB-4 at follow-up.

We further compared baseline characteristics between participants classified as false negatives (n = 9) and true negatives (n = 120). Demographic and metabolic characteristics, including age, sex, BMI, race, diabetes duration, HbA1c, insulin/glucagon-like peptide-1 use, and metabolic syndrome prevalence, were similar between groups. However, false-negative participants had significantly higher baseline AST (median 39 vs 23.5 U/L, *P* = .025) and ALT (median 40 vs 25 U/L, *P* = .012), as well as higher baseline liver stiffness on VCTE (10.8 vs 5.6 kPa, *P* < .0001) and MRE (4.1 vs 2.3 kPa, *P* = .0003).

### Sensitivity Analysis

We evaluated applying a lower FIB-4 cut point of 1.0 to define low risk, where a FIB-4 of 1.0 to 2.67 would fall in the indeterminate risk range (Supplementary Figure 1). The number of false negatives decreased from 9 to 3 at baseline and 3 to 0 at follow-up. However, the number of patients requiring VCTE increased from 67 (42.1%) to 112 (70.4%) at baseline and 58 (45%) to 80 (69.7%) at follow-up. Referral to specialty care was 24.5% at baseline and 8.7% at follow-up (Table 4). We also evaluated a lower MRE threshold of 3.14 kPa to define significant fibrosis, in alignment with recent American Association for the Study of Liver Diseases guidelines and data supporting MRE liver stiffness measurement of 3.1 to 3.5 kPa compatible with at least significant fibrosis.^18^^,^^27^ Using this lower threshold at baseline and 2-year follow-up resulted in a false negative rate of 10.9% (low risk but with significant fibrosis) at baseline, and 4.4% at 2-year follow-up.

**Table 4:** Comparison of AGA Clinical Pathways

	Patients referred to specialty care	False negatives
AGA pathway at baseline	28 (17.6%)	9 (7.0%)
AGA pathway after 2-y assessment	9 (7.0%)^a^	3 (3.0%)
AGA pathway at baseline with FIB-4 >1.0	39 (24.5%)	3 (3.0%)
AGA pathway after 2-y assessment with FIB-4 >1.0	10 (8.7%)^a^	0 (0.0%)

a Additional number of patients after 2-y assessment.

## Discussion

In this prospective longitudinal study of adults with T2DM, we demonstrate that repeat application of the clinical care pathway over a 2-year interval decreases false negatives from 7% to 3% with only a small increase in referral to specialty care. Most patients who were initially missed or incorrectly classified as low risk at baseline were done so by FIB-4 assessment. These patients had baseline FIB-4 values between 1.0 and 1.3 yet had significant fibrosis on MRE. Lowering the FIB-4 cutoff to 1.0 eliminated all false negatives but increased downstream VCTE use by 54% more over 2-years. Using a more conservative MRE cut point of 3.14 kPa led to a significant increase in the number of false negatives at baseline (10.9%) but was reduced to 4.4% after 2-year follow-up assessment. Collectively, our findings confirm the longitudinal utility of this clinical pathway, with fewer false negatives over time, while highlighting that lowering the FIB-4 threshold could improve detection of fibrosis when weighed against the added resource burden of increased VCTE utilization.

### In Context With Published Literature

Several studies have now demonstrated that NITs are directly associated with the risk of liver-related events, specifically looking at FIB-4 index,^28^^,^^29^ VCTE,^30^^,^^31^ MRE,^32^, ^33^, ^34^ or a stepwise FIB-4/VCTE algorithm.^35^ In a prospective cohort study including 12,950 participants from the United States, Asia, and Europe, it was shown that the two-step strategy of FIB-4/VCTE stratifies future liver-related events across risk tiers (5-year cumulative incidence 0.5%, 1.0%, and 10.8% in low-, indeterminate-, and high-risk groups), underscoring the pathway’s prognostic value.^36^ However, this analysis did not evaluate the longitudinal use of the algorithm to detect patients with significant fibrosis, who may qualify for and benefit from liver-directed treatment.

The impact of serial FIB-4 measurements had been investigated in a large population-based study, which demonstrated that transitions from low or indeterminate to high FIB-4 categories over 5 years were associated with an 8-fold increased risk of severe liver outcomes.^37^ Similarly, longitudinal VCTE data show that increases in liver stiffness on repeat testing correlate with future liver-related events.^38^ However, the outcomes of these studies were not focused on the performance of multistep NIT-based screening strategies. As highlighted in a consensus report by the American Diabetes Association,^39^ and recommended per the AGA Clinical Practice Update,^40^ systematic implementation of longitudinal screening strategies in high risk patients (eg, with T2DM) is essential to accurately identify MASLD early and to assess for disease progression/regression.

Our findings build on and extend recent validation efforts of the AGA pathway. In a cross-sectional study including 2322 participants in the United States, there was found to be limited PPV when using VCTE as the reference, an approach that cannot fully evaluate the pathway given VCTE is an embedded step.^41^ While liver biopsy remains the reference standard, it would be unethical to perform across the full study cohort, particularly those with low probability of significant fibrosis. In contrast, MRE was used as the reference in prospectively studied adults with T2DM to demonstrate that the AGA pathway maintained a low false-negative rate (<5%) for advanced fibrosis while substantially limiting specialty referrals in a cross-sectional study.^19^ Moreover, a prior study including individuals with metabolic dysfunction and alcohol-associated liver disease also demonstrated a good performance of this clinical pathway, with a false negative rate of 2% for significant fibrosis.^42^ Our study advances this work by evaluating the pathway longitudinally in patients with MRE at baseline and follow-up, showing that repeat interval assessment further reduces false negatives in patients with significant fibrosis.

### Strengths and Limitations

The use of MRE as the reference standard for fibrosis assessment is a key strength, as it provides superior accuracy and reproducibility compared with serum-based or elastography-only methods and represents the most accurate noninvasive imaging biomarker currently available. However, MRE also may misclassify a subset of patients (sensitivity and specificity of 87% and 88%, respectively, for advanced fibrosis^18^), with potential misclassification affecting our results. Regardless, liver stiffness on MRE is directly associated with future liver-related events, supporting its use in this context.^32^ Second, the prospective and longitudinal design with 2-year follow-up allowed for repeat evaluation of risk classification, demonstrating that the clinical pathway retains validity over time and that false-negative rates diminish with serial testing, an important insight for real-world clinical practice. A limitation of the study is the relatively short follow-up period, which may not adequately capture long-term outcomes such as cirrhosis or liver-related events. However, this was not one of the outcomes of this study, and this interval period was used as it is suggested by current guidelines. Another limitation is that the study was performed in patients with T2DM, whereas the pathway can be applied to other populations with 2 or more metabolic risk factors or steatosis or elevated aminotransferases. In addition, the study population was restricted to adults aged 50 years and greater, which may limit generalizability to younger individuals with T2DM who may have different fibrosis risk profiles. However, T2DM has been identified as a key risk factor for disease progression, making performance in this studied population of significant clinical importance. We did not evaluate longitudinal changes in glycemic control or other measures of diabetes severity over the follow-up period. Although baseline HbA1c was available, glycemic control is dynamic and may influence fibrosis progression and pathway performance over time. We acknowledge that although baseline characteristics were largely comparable between participants with and without follow-up, individuals who did not complete follow-up were more likely to be Hispanic and less likely to be non-Hispanic White. This differential retention may limit generalizability of longitudinal findings across racial and ethnic groups; however, because baseline fibrosis measures and metabolic risk factors were similar between groups, substantial attrition bias affecting outcomes is less likely. In addition, a small number of participants (7/626, 1.1%) had invalid VCTE measurements and were excluded, which could introduce selection bias; however, given the minimal proportion affected, the impact on study findings is likely negligible. Finally, this was a single-center study, which could limit generalizability; nonetheless, the demographic diversity of our cohort enhances applicability to broader populations and provides a foundation for future multicenter validation.

## Implications for Clinical Practice and Future Research

Our findings have direct application for clinical practice, providing granular support of the current guidelines for risk stratification among individuals aged ≥50 years with T2DM. However, the optimal interval for reassessment requires further clarification. Moreover, studies should evaluate the economic and clinical impact of such modifications and explore integration of individualized risk factors (eg, obesity, ALT elevation, genetic risk, age-adjusted cutoffs) into pathway refinements.^43^

## Conclusion

This study validates the longitudinal performance of the clinical pathway endorsed by major academic societies in individuals with T2DM. Adherence to current guideline recommendations for reassessment every 2 to 3 years resulted a decrease in false negatives, with most missed baseline cases reclassified appropriately on follow-up. Lowering the FIB-4 cutoff further reduced false negatives, but at the expense of increased downstream testing, raising important questions of cost-effectiveness and resource allocation.

## Supplementary Materials

Supplementary Figure 1 Performance of AGA Clinical Pathway in adults with T2DM with MRE as the reference, at baseline and at 2-year Follow-up, with lower FIB-4 cut point of 1.0

Supplementary Table 1 Clinical, demographic, and imaging characteristics of participants with follow-up and those without follow-up.

Extended PDF
