# Supplementary material for: Longitudinal Validation of Clinical Care Pathways for Metabolic Dysfunction–Associated Steatotic Liver Disease in a Prospective Cohort of Individuals With Type 2 Diabetes
Source: Gastro Hep Adv. 2026 May 27;5(9):101022. doi: 10.1016/j.gastha.2026.101022 (PMC13332004; doi:10.1016/j.gastha.2026.101022)
Supplement: Supplementary Figure 1 — Performance of AGA Clinical Pathway in adults with T2DM with MRE as the reference, at baseline and at 2-year Follow-up, with lower FIB-4 cut point of 1.0 [file mmc1.pdf]

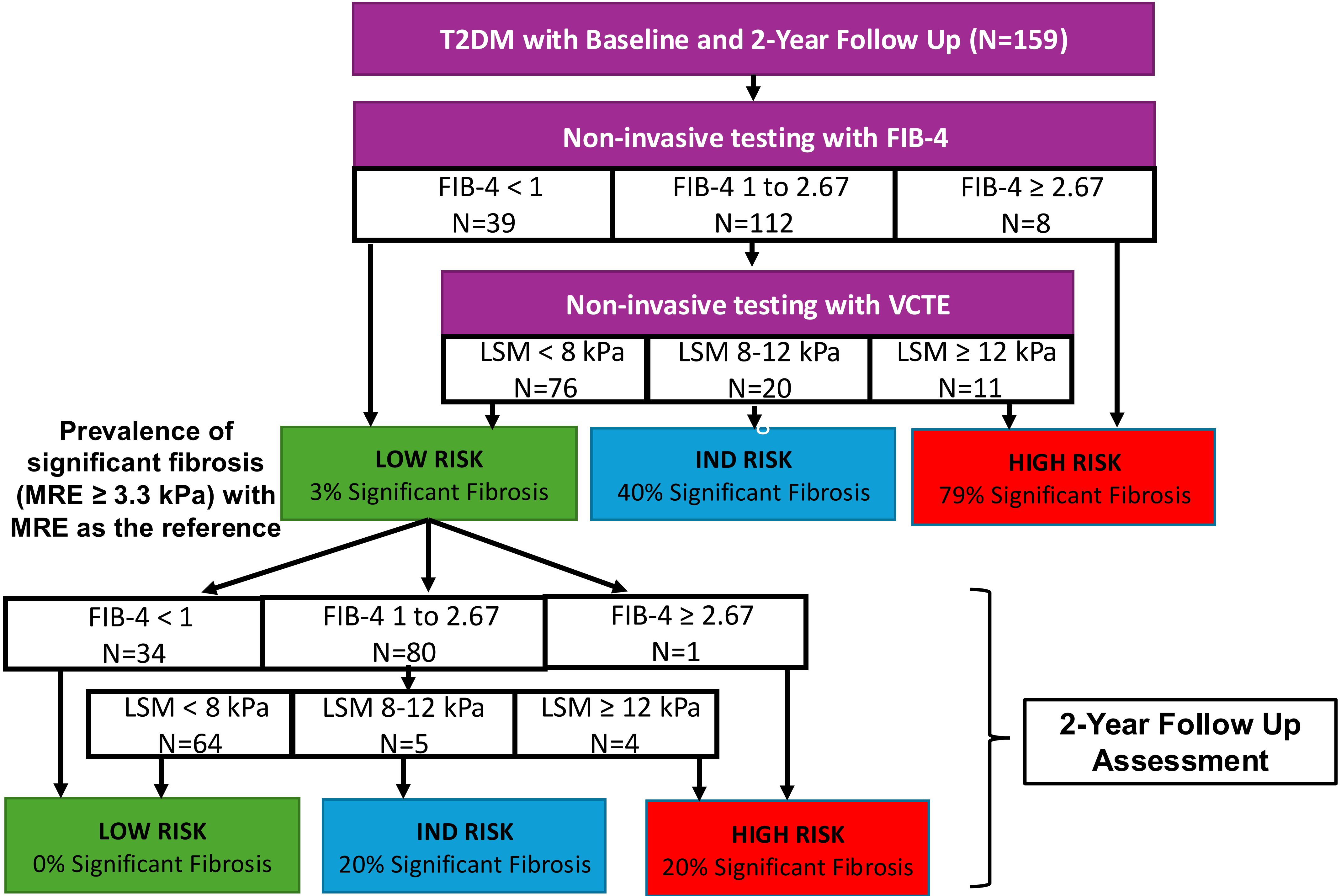
T2DM with Baseline and 2-Year Follow Up (N=159)
Non-invasive testing with FIB-4
FIB-4 < 1
N=39
FIB-4 1 to 2.67
N=112
FIB-4 ≥ 2.67
N=8
Non-invasive testing with VCTE
LSM < 8 kPa
N=76
LSM 8-12 kPa
N=20
LSM ≥ 12 kPa
N=11
Prevalence of significant fibrosis (MRE ≥ 3.3 kPa) with MRE as the reference
LOW RISK
3% Significant Fibrosis
IND RISK
40% Significant Fibrosis
HIGH RISK
79% Significant Fibrosis
FIB-4 < 1
N=34
FIB-4 1 to 2.67
N=80
FIB-4 ≥ 2.67
N=1
LSM < 8 kPa
N=64
LSM 8-12 kPa
N=5
LSM ≥ 12 kPa
N=4
LOW RISK
0% Significant Fibrosis
IND RISK
20% Significant Fibrosis
HIGH RISK
20% Significant Fibrosis
2-Year Follow Up Assessment
