# Supplementary material for: Longitudinal Validation of Clinical Care Pathways for Metabolic Dysfunction–Associated Steatotic Liver Disease in a Prospective Cohort of Individuals With Type 2 Diabetes
Source: Gastro Hep Adv. 2026 May 27;5(9):101022. doi: 10.1016/j.gastha.2026.101022 (PMC13332004; doi:10.1016/j.gastha.2026.101022)
Supplement: Supplementary Table 1 — Clinical, demographic, and imaging characteristics of participants with follow-up and those without follow-up. [file mmc2.docx]

**Supplemental Table 1.** Clinical, demographic, and imaging characteristics of participants with follow-up and those without follow up.

|  | **Baseline Assessment Only (N=417)** | **Baseline and Follow up (N=209)** | **P** |
| --- | --- | --- | --- |
| **Demographic and Clinical** |  |  |  |
| Age in years, mean (SD) | 63.9 (8.1) | 64.3 (7.9) | 0.5258 |
| Female, n (%) | 272 (65.2%) | 126 (60.3%) | 0.2257 |
| BMI (kg/m^2^), mean (SD) | 31.8 (6.6) | 31.2 (4.9) | 0.1434 |
| Obesity (BMI ≥ 30 kg/m^2^) | 238 (57.2%) | 125 (59.8%) | 0.5347 |
| Race |  |  | **<0.0001** |
| White, n (%) | 126 (30.8%) | 101 (49.3%) |  |
| Hispanic, n (%) | 192 (46.9%) | 61 (29.8%) |  |
| Asian, n (%) | 64 (15.7%) | 30 (14.6%) |  |
| Other, n (%) | 27 (6.6%) | 13 (6.3%) |  |
| Duration of DM (years), median (IQR) | 8 (12.5) | 8 (12) | 0.6651 |
| Hypertension, n (%) | 257 (61.6%) | 130 (62.2%) | 0.8898 |
| Hyperlipidemia, n (%) | 216 (51.8%) | 123 (58.9%) | 0.0949 |
| Metabolic Syndrome, n (%) | 246 (64.9%) | 119 (65.8%) | 0.8456 |
| **Biochemical profile** |  |  |  |
| HbA1c (%), median (IQR) | 6.8 (1.6) | 6.8 (1.5) | 0.2594 |
| HOMA-IR | 5.1 (5.6) | 4.8 (5.3) | 0.6692 |
| AST (U/l), median (IQR) | 25 (13) | 26 (17) | 0.1148 |
| ALT (U/l), median (IQR) | 25 (21.5) | 29 (25) | 0.0263 |
| Alkaline Phosphatase (U/l), median (IQR) | 81 (33) | 76 (34) | 0.0148 |
| Total bilirubin (mg/dl), median (IQR) | 0.5 (0.3) | 0.5 (0.2) | 0.9543 |
| Albumin (g/dl), median (IQR) | 4.4 (0.3) | 4.5 (0.3) | 0.4467 |
| Triglycerides (mg/dl), median (IQR) | 137 (91) | 143 (80) | 0.1195 |
| HDL (mg/dl), median (IQR) | 46 (16) | 45.5 (16) | 0.3110 |
| LDL (mg/dl), median (IQR) | 86 (44) | 84 (45) | 0.5797 |
| Platelet count (10^9^/L), median (IQR) | 242 (82) | 245 (84) | 0.6199 |
| INR, median (IQR) | 1 (0.1) | 1 (0.1) | 0.8008 |
| **Blood based scores and markers** |  |  |  |
| FIB-4, median (IQR) | 1.3 (0.8) | 1.3 (0.8) | 0.4391 |
| NAFLD Fibrosis Score, median (IQR) | -0.3 (1.5) | -0.4 (1.5) | 0.4973 |
| **Imaging** |  |  |  |
| CAP (dB/m), mean (SD) | 304.6 (58.0) | 313 (52.2) | 0.0810 |
| VCTE (kPa), mean (SD) | 7.1 (5.5) | 7.9 (6.9) | 0.1365 |
| MRI-PDFF (%), mean (SD) | 9.8 (8.1 | 10.5 (7.7) | 0.2978 |
| MRE (kPa), mean (SD) | 2.6 (1.1) | 2.8 (1.3) | 0.2219 |

Abbreviations: HbA1c, Hemoglobin A1c; AST, Aspartate aminotransferase; ALT, Alanine aminotransferase; BMI, Body mass index; HDL, high-density lipoprotein; INR, International normalized ratio; IQR, interquartile range; LDL, low-density lipoprotein; FIB-4, Fibrosis index based on the 4 factor; MRI-PDFF, MRI-based proton density fat fraction; MRE, Magnetic resonance elastography; SD, Standard deviation. T-test performed on continuous variables presented as mean (SD), Wilcoxon rank sum test performed on all other continuous variables. Significant fibrosis defined as MRE ≥ 3.30 kPa.
